# Multi-omics profiling unravel the immune landscape diversity by prognostic signatures of immunotherapy response in triple-negative breast cancer

**DOI:** 10.3389/fimmu.2025.1735893

**Published:** 2025-12-18

**Authors:** Rong Chai, Ziting Zhang, Zheng Gong, Qi Li, Chunyan Dong

**Affiliations:** Department of Oncology, Shanghai East Hospital, School of Medicine, Tongji University, Shanghai, China

**Keywords:** immunotherapy, prognostic signature, scRNA-sequencing, TNBC, tumor immune infiltration

## Abstract

**Background:**

Triple-negative breast cancer (TNBC) remains a challenging malignancy with limited therapeutic options and variable responses to immune checkpoint inhibitors (ICIs). The tumor immune infiltration significantly influences the outcomes of immunotherapy treatments. Novel biomarkers are urgently needed which integrate both tumor-intrinsic and immune-related features to better stratify patients and dissect the immune microenvironment.

**Methods:**

We investigated the tumor immune infiltration and assessed its prognostic significance in an internal cohort of TNBC patients using multiplex immunofluorescence. Then we integrated multi-omics approach that combines bulk and single-cell RNA sequencing to develop a prognostic signature. The model underwent validation across three independent external cohorts and additional immunotherapy cohorts. Immune cell infiltration was assessed using CIBERSORT, and cellular communication networks were characterized through CellChat analysis. Expression and functional investigations of key genes were conducted in TNBC cell lines using knockdown and overexpression techniques and further functional assays.

**Results:**

Our internal cohort of patients with TNBC revealed distinct TIME profiles and both high CD8^+^ T cell density (HR = 0.22, 95%CI: 0.05-0.92, P = 0.0164) and low Treg density (HR = 5.836, 95%CI: 1.60-21.37, P = 0.0004) were independently associated with improved overall survival. Integrated characterization of tumor and immune features, a four-gene prognostic signature comprising CD276, MS4A1, IGFBP1, and CD200 was established. The signature categorized TNBC patients into distinct risk strata exhibiting varied survival outcomes and distinguished tumor immune infiltration conditions. The low-risk group exhibited enhanced immune infiltration, effector T cell activity, and favorable responses to ICIs therapy. Conversely, the high-risk group showed an immunosuppressive microenvironment. Immunofluorescence revealed a spatial association and potential functional interplay between MS4A1, CD200 and CD8^+^ T cells. *In vitro* researches demonstrated that CD276 enhances cell growth and migration, whereas IGFBP1 exerts protective effects.

**Conclusions:**

We developed and validated an immune-related signature for predicting TNBC outcomes and immunotherapy response. This signature reflects underlying immune landscape heterogeneity and provides a crucial method for patient stratification and immunotherapeutic planning.

## Introduction

The absence expression of estrogen and progesterone receptors (ER\PR) as well as human epidermal growth factor receptor 2 (HER-2) defines triple-negative breast cancer (TNBC) in molecular perspective, which narrows down receptor targeted therapeutic choices and leads to its unfavorable prognosis (1–3). However, TNBC exhibits a propensity to be responsive to immunotherapies, particularly when the tumor displays a high TMB (tumor mutational burden) and PD-L1 levels while abundant lymphocytes infiltrating—all indicators of an immunogenic tumor microenvironment (4, 5). Therefore, the ICI (immune checkpoint inhibitor) therapy which intervenes in the immunosuppressive process and activates tumor immunity, has drastically changed treatment paradigm of TNBC (6, 7). Key clinical trials have been instrumental in establishing ICIs as part of the standard treatment paradigm. The IMpassion130 study showed improved survival outcomes in metastatic TNBC who received atezolizumab in conjunction with nab-paclitaxel, particularly in cases where tumors exhibited PD-L1 positivity (8). Similarly, the KEYNOTE-355 trial revealed prolonged survival in advanced TNBC patients whose up-regulation of PD-L1 was contraposed by pembrolizumab in combination with chemotherapy (9–11).

Although ICIs have dramatically reshaped therapeutic strategies for TNBC, the strong heterogeneity of TNBC leads to unsatisfactory efficacy of immunotherapy (12). A large fraction of patients develop intrinsic or adaptive resistance, reducing the effectiveness of ICIs (13). It is estimated that only 20-30% of TNBC patients achieve a sustained response to ICIs, highlighting the necessity for improved patient stratification strategies (14). Current biomarkers like PD-L1 levels, TILs, and TMB have shown restricted predictive accuracy and clinical utility. For instance, PD-L1 positivity is not always indicative of response, and TIL evaluation can be methodologically variable and context-dependent (15–19). Similarly, TMB, although useful in some cancer types, exhibits only modest predictive value in breast cancer (20). These limitations highlight the critical requirement for more biologically relevant biomarkers to better guide immunotherapy decisions and optimize results for TNBC.

The type and number of tumor-infiltrating immune cells, also called the tumor immune microenvironment (TIME) are closely related to the response to immunotherapy (21). Especially, the presence and functional state of effector T cells are particularly critical, as they directly mediate the killing of tumor cells and are strongly associated with positive treatment outcomes (22–24). There is growing recognition that effective biomarkers should reflect both tumor-intrinsic features and immunity-tumor crosstalk (25). Genes related to immune regulation may serve as ideal candidates for constructing such biomarkers, given their central role in shaping antitumor immunity and influencing clinical outcomes.

In our study, internal cohort of patients with TNBC revealed distinct TIME profiles. Patients with more infiltrated CD8 + T cell had better overall outcome, while more Treg cells correlated with worse one.

**Figure 1:**
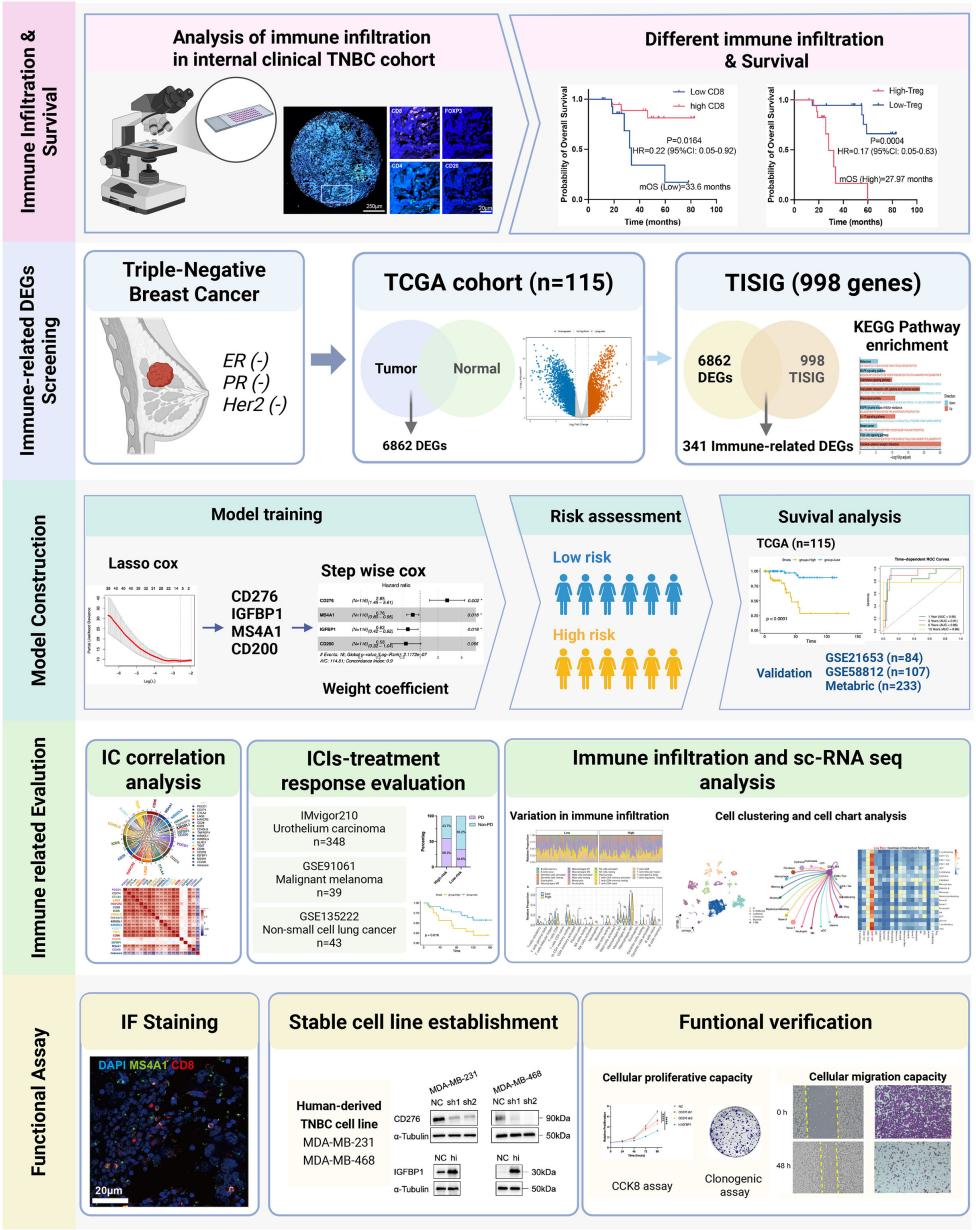
The graphic scheme of this research.

And we created and validated an immune-related prognostic model that integrates both tumor biological behavior and immune contexture to forecast patient outcomes and the immune landscape in TNBC. Using transcriptomic information from The Cancer Genome Atlas (TCGA), TISIDB (a gene set for the interaction between T cells and tumor cells validated by high-throughput data),as well as other available cohorts, we identified a four-gene signature consisting of CD276, MS4A1, IGFBP1, and CD200 (26). We further validated the prognostic and predictive value of this signature across multiple independent cohorts and explored its biological underpinnings through immune infiltration analysis, single-cell RNA sequencing, and *in vitro* functional validations. Our study delivers a risk-stratification tool and uncovers immune-related factors of the heterogeneous responses observed in TNBC. **Figure 1** outlines the overall experimental design.

## Materials and methods

### Multiplex immunofluorescence staining and analysis

We established an internal cohort by collecting tumor tissues and follow-up data from 38 TNBC patients. Paraformaldehyde-fixed tissues were used for paraffin embedding and made into sections. All patients involved in tissue donation signed informed consent and were reviewed by the Ethics Committee (Number: Csyayj2024098). Multiplex immunofluorescence was performed using the Opal™ 5-Color Manual IHC Kit followed instructions. The prepared paraffin sections undergo deparaffinization, rehydration, antigen retrieval through heating, and blocking of peroxidase activity (as well as nonspecific binding blocking). The sequential staining protocol involved repeated cycles of primary antibody incubation, tyramide signal amplification (TSA) with Opal fluorophores, and microwave-assisted antibody stripping. After all staining cycles were completed, slides were mounted.

CD8^+^ T cells and FOXP3^+^ T regulation cells’ densities were calculated as positive cells per square millimeter in the total area. Based on these densities, patients were stratified into “immune-activated” and “immune-suppressed” subgroups.

### Data collection

We gathered clinical and sequencing data from the TCGA-TNBC cohort (comprising 115 cases) and matched normal breast tissue samples (113 cases) through the TCGA-BRCA Project’s repository (available at https://portal.gdc.cancer.gov/). To verify our findings, we pulled RNA-seq datasets from four GEO series (GSE21653, GSE58812, GSE91061, and GSE135222) along with scRNA-seq database from GSE176078—all available through GEO repository. These resources served as the foundation for our analytical validation (27, 28). Additionally, The METABRIC dataset was sourced from CBIOPORTAL website (http://www.cBioPortal.org/). IMvigor210 datasets acquired from IMVIGOR210CoreBiologies data package. Above data sets were analyzed in Rstudio software. Nine hundred and ninety-eight TISIDB-related genes (TISIGs) were downloaded from http://cis.hku.hk/TISIDB/index.php.

### Analysis of DEGs

To pinpoint the genes with differential expression patterns between TNBC tumors and healthy tissues, we employed “limma” package (29). Cutoff value of this step were set at Adjusted P-Value ≤0.05 and | log2 fold change | ≥1.We used TPM-normalized data and applied the removeBatchEffect function in limma to correct for batch effects. With R package “clusterProfiler”, DEGs were used for KEGG pathway enrichment analysis (30). By intersecting DEGs with TISIGs, three hundred and forty-one immune-related DEGs were identified.

### Identification of risk genes and construction of prognostic signature

Prognosis related four genes (CD276, IGFBP1, MS4A1, CD200) were confirmed through Lasso regression by investing 341 TISI-related DEGs (31). Stepwise multivariate Cox regression was employed to establish an immune-related prognostic signature, which was calculated by risk score formula: 1.047*CD276 - 0.470*IGFBP1 - 0.281*MS4A1 - 0.548*CD200. Using the formula above, each sample received a risk score and were divided into two distinct risk strata with the TCGA-TNBC cohort’s median value.

To evaluate our signature’s predictive capacity, receiver operating characteristic (ROC) curve was run to determine its forecasting performance at 1, 3, 5, and 10-year intervals. Additionally, survival analysis assessed outcome variations across risk groups (32).

### Immunotherapy response analysis

By introducing major ICIs, immune-activating costimulatory molecules, and NK cell-related immune checkpoints, coefficients of correlation linking these immune checkpoint molecules to the prognostic signature were calculated.

Immunotherapy-related transcriptome sequencing data and clinical prognostic data including immunotherapy data for urothelial carcinoma (IMvigor210, n = 348), melanoma (GSE91061, n = 43), and non-small-cell lung carcinoma (GSE135222, n = 39), were used to assess the prognostic signature’s predictive accuracy. After calculating individual risk scores, cohort members were stratified into two risk categories according to the median cutoff. Survival analysis assessed variations in prognosis. And percentages of PD versus non-PD patients in both groups after immunotherapy were calculated.

### Immune landscape analysis

Immune scores, stromal scores, and tumor purity for each sample were computed using the “estimate”, to deepen insights into the correlation between TME and predictive models (33).

The CIBERSORT algorithm assessed immune cell composition across the two sample groups, assessing both the correlation of individual cell types with specific risk genes and identifying statistically significant variations in cellular composition between the groups (34).

### Single-cell RNA sequencing and CellChat analysis

Based on R package “Seurat”, TNBC single-cell transcriptome data from dataset GSE176078 (n=10) was used to validate the ability of prognostic models and explore cell-cell interactions further (35). Cells with fewer than 299 expressed genes (bottom 2%), over 5716 expressed genes (top 2%), or 10 or more mitochondria-related genes were removed as abnormal cells. After normalizing the Seurat object and searching for hypervariable genes. PCA was conducted, selecting the top 30 components with a resolution of 0.1. Additionally, we applied UMAP-based dimensionality reduction through the RunUMAP function. Cells were categorized into six groups by marker gene expression. Using the Z-score method to standardize risk scores calculated for each type of cell, patients were then categorized according to their individual scores. At the same time, through the Featureplot function, the expression of every single risk gene in all cells was displayed.

In order to further refine the cell population and prepare for cell-to-cell communication analysis, immune cells, encompassing T/NK, B and myeloid cells, were further classified into subgroups. Specific types of cell subsets were extracted from Seurat object containing all cells, and dimension reducing and clustering were re-performed. The initial 30 principal components were chosen with a resolution of 0.2. Then, all sorted subpopulation cells were mapped back to the main Seurat object that contains all the cells for further CellChat analysis.

Using the “cellchat” R package, an analysis of intercellular communication was performed. And comparisons were made to identify variations in the intensity of interactions among various cellular subsets. Meanwhile, the expression intensity of various signaling pathways in different subpopulations was also visualized.

### Selection and establishment of cell lines

Both kinds of TNBC cells, MDA-MB-231 and MDA-MB-468, were maintained with L15 complete medium (Gibco), while DMEM complete medium (Gibco) for 293T. Both media contained ten percent FBS and a one percent penicillin-streptomycin solution (Gibco) to maintain optimal growth conditions.

### Construction and validation of transgenic cell lines

The construction of IGFBP1 overexpressed plasmids was dependent on cytomegalovirus plasmids, while knockdown plasmids of CD276 was dependent on lentiviral vector plasmids. All shRNA sequences of knockout plasmid were picked up from the Merck website. After waiting for 293T cells to enter a logarithmic state of proliferation, the mixed target plasmid, tool plasmid and transfection agent were added to culture medium of 293T cells. The supernatant of 293 T cells was harvested after 48 hours as the viral solution combined with 10 μg/mL of polybrene added to the target cells. After another 48 hours, corresponding antibiotics were added to infected cells and control groups according to the resistance gene on the target plasmids. As cells of control groups were wiped out, remaining infected cells were those whose genes had been successfully manipulated. Overexpression or knockout of target genes was examined by Western Blot assay. The shRNA sequences:

sh1 5’-CAAAGAAGATGATGGACAAGA-3’;

sh2 5’-GCTTGTTTGATGTGCACAGCA-3’.

### CCK-8 assay

First, seed the different stable transfected cell lines in a 96-well plate (1,000 cells/well) and culture them in an incubator (37°C, 5% CO_2_) varying durations of 0–4 days. At each time point, add CCK-8 assay kit (10μL, CK04, Dojindo, Japan) to the wells and continue to incubate for two hours in light shielding condition. Then, absorbance of 450 nm light was measured by microplate reader (Biotek Eon, USA) for analysis.

### Colony formation assay

TNBC cells were seed in six-well plates (1 × 10^3^ cells/well) and incubated for a span of 14 days. Following this, the plates were gently washed and then treated by 4% paraformaldehyde (15 mins). They were then stained with crystal violet (10 mins). Once this step was complete, the colonies were carefully washed with deionized water and pictured for detailed analysis. To count the colonies, the ImageJ software (version 1.54) was subsequently used.

### Wound‐healing scratch assay

Six-well plates were used to culture cells until they achieved nearly complete monolayer coverage (90% confluency). A 200-μL pipette tip, sterilized, was applied to produce consistent scratches, followed by a delicate rinsing of the wells with PBS. The cultures were subsequently maintained in serum-deprived medium for a 48 h. Wound site images were taken at baseline (0 h) and post-incubation (48 h) for comparison. The migration rate was then quantified using ImageJ software.

### Cell migration assay

Migratory potential of cells was assessed in 24-well system featuring 8.0 μm polycarbonate filters (Corning, USA). Briefly, we seeded 10^5^ cells into 200 μL of medium (without serum) and transferred them to the chamber. The wells held 600 μL of the full medium (including 10% FBS), and cultured for 48 h (37°C, 5% CO_2_). Post-incubation, we carefully wiped away the rest cells on top side of membrane with a cotton swab. Crossed cells were treated with 4% paraformaldehyde for 15 minutes, followed with crystal violet staining. Then, we carefully cut out the membrane and mounted it on a microscope slide. Using an inverted microscope (Axiovert A1-FL, courtesy of Zeiss, Germany), we captured images of the cells for further study.

### Statistical analysis

Most data processing and statistical analyses were accomplished with R and GraphPad Prism (v10.3.0). R packages used in Bioinformation analysis: cBioPortalData, survival, survminer, TCGAbiolink, dplyr, ggplot2, biomaRt, limma, ggrepe, glmnet, MASS, clusterProfiler, org.Hs.eg.db, pheatmap, rms, timeROC, pROC, CIBERSORT, tidyr, ggpubr, RColorBrewer, estimate, tidyverse, ComplexHeatmap, corrplot, circlize, GGally, Hmisc, circlize, RColorBrewer, Matrix, SeuratObject, Seurat, reticulate, cowplot, magrittr, patchwork, scater, clustree, stringr, harmony, CellChat.

We utilized the Student’s t-test for analyzing normally distributed data within two groups and the Wilcoxon rank-sum test for evaluating non-parametric distributions. KM survival analysis was conducted, with the log-rank test assessing group disparities. The significance threshold was established at P<0.05.

## Results

### Distinct TIME profiles and survival in internal cohort

**Figure 2:**
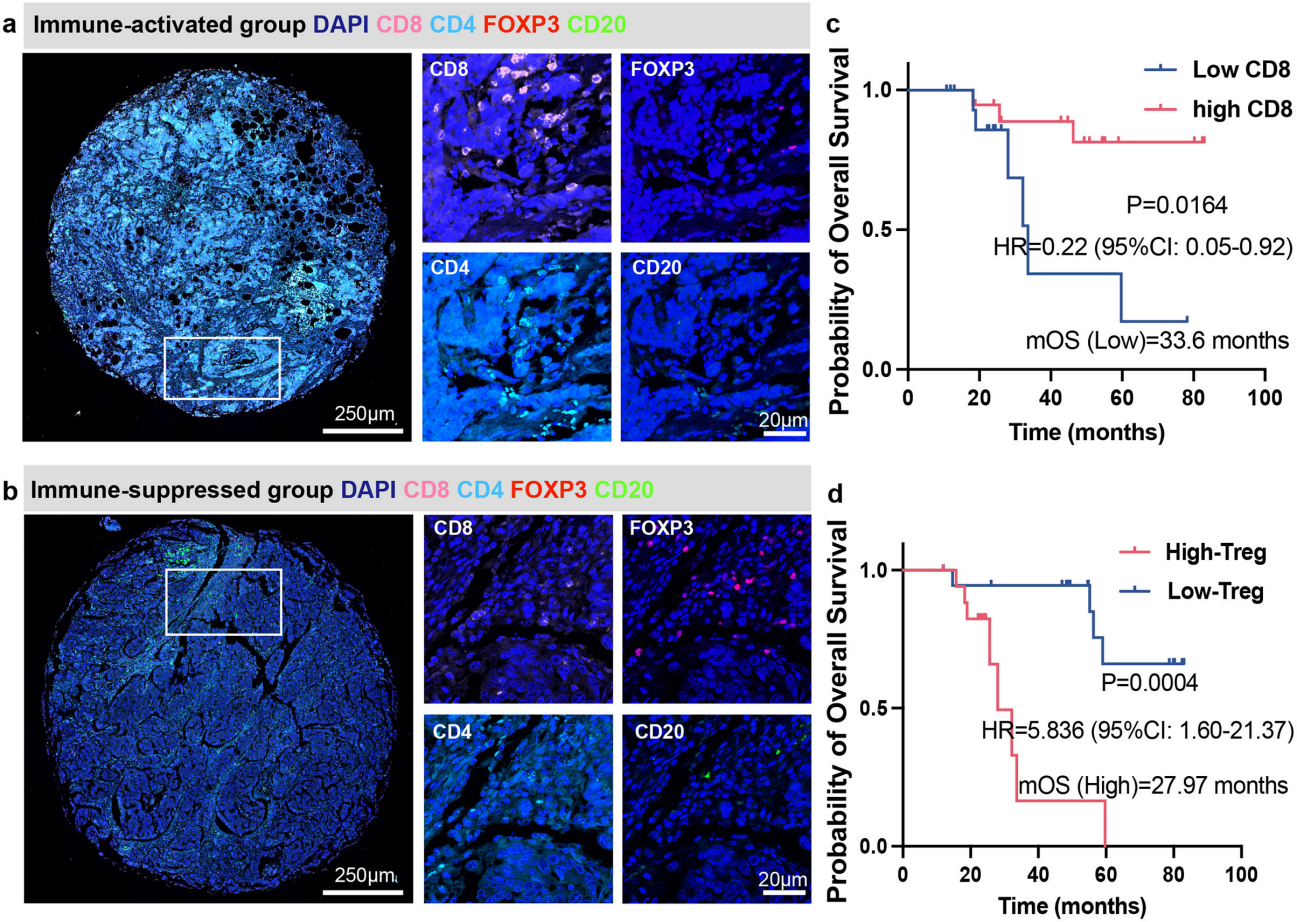
Immune infiltration and survival in internal patient cohort. **(a, b)** Multiplex immunofluorescence staining of immune-activated group and immune-suppressed group. **(c, d)** Survival analysis between high- and low-CD8 (Log-rank test, HR = 0.22, 95% CI: 0.05-0.92, p = 0.0164) or Treg groups (Log-rank test, HR = 5.84, 95% CI: 1.60-21.37, p = 0.0004) based on patients’ overall survival (months).

In our internal cohort of patients with TNBC, multiplex immunofluorescence staining revealed distinct TIME profiles (**Figures 2a, b**). Patients were dichotomized into an immune-activated group, featuring abundant CD8^+^ and CD4^+^ T cell infiltration with minimal Tregs, and an immune-suppressed group, dominated by Tregs with reduced CD8^+^ and CD4^+^ T cells. Furthermore, stratification based on median levels of CD8^+^ and Treg infiltration demonstrated that high CD8^+^ T cell density (HR = 0.22, 95%CI: 0.05-0.92, P = 0.0164) independently attach improved outcome to overall survival, which is also applicable to low Treg density (HR = 5.836, 95%CI: 1.60-21.37, P = 0.0004) (**Figures 2c, d**). While this internal cohort served for initial hypothesis generation through immune phenotyping, the subsequent prognostic model was built and validated using large, independent transcriptomic datasets.

### Immune‐related prognostic signature construction

**Figure 3:**
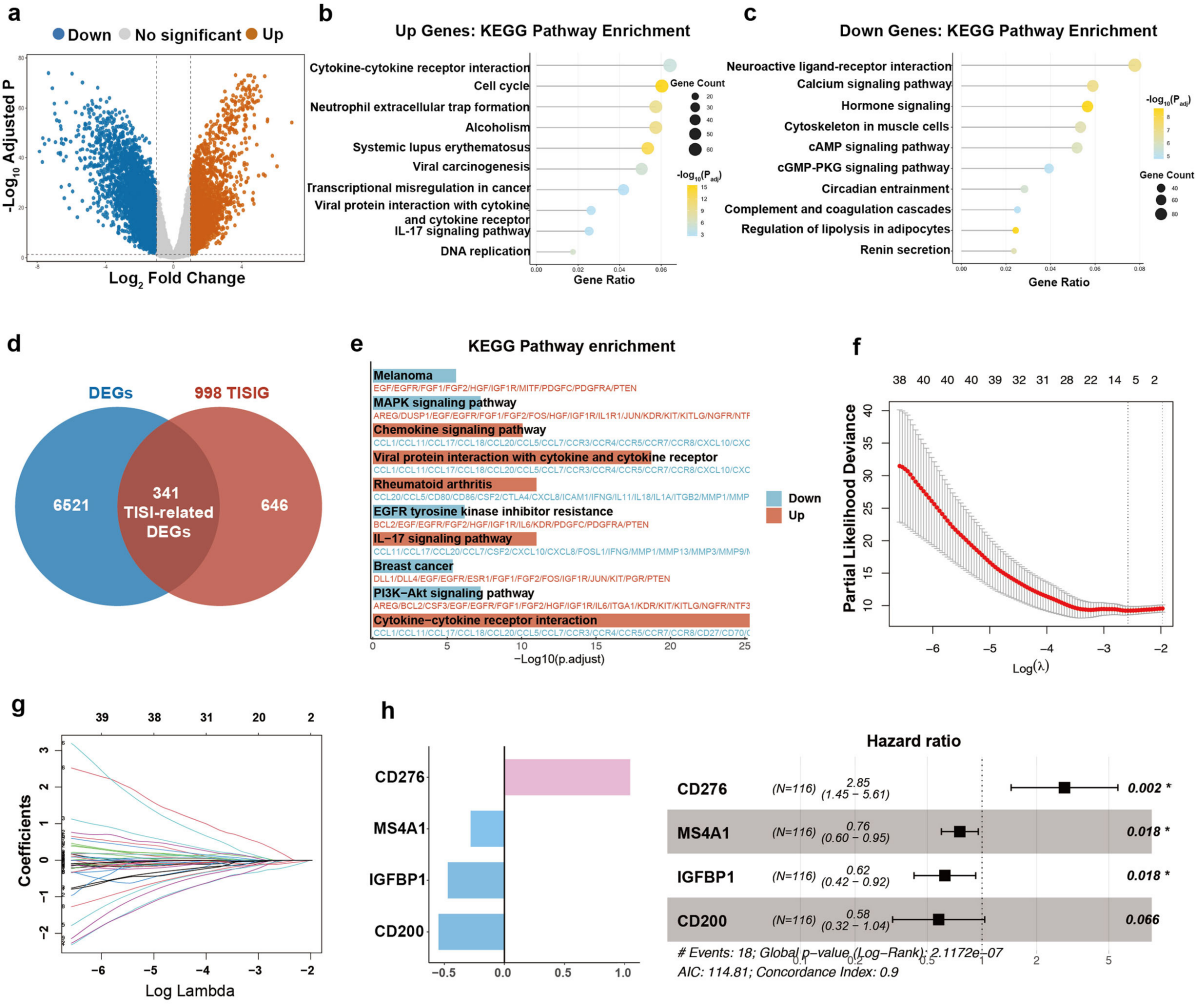
Immune‐related prognostic signature construction. **(a)** Volcano plot shows differential expressed genes of TCGA-TNBC tumor tissues compared with normal tissues. Cutoff value:log2 fold change≥1 and adjusted P. Value ≤0.05. **(b, c)** Top 10 KEGG pathways of up- and down-regulated genes according to DEGs result. **(d)** Venn diagram of the DEGs and the TISIDB immune-related genes (TISIG). **(e)** Top 10 KEGG pathways of enrichment analysis based on 341 TISI-related DEGs. **(f, g)** The coefficient paths of each independent variable across lambda values and their initial distributions. **(h)** Forest plot of four candidate risk genes, and the bar graph shows risk coefficients.

In the TCGA-TNBC cohort, We identified 6862 DEGs between tumor and healthy tissues of 115 TNBC patients (adjusted P<0.05 and |log_2_FC|>1). Compared with normal tissues, tumor tissues exhibited 2,809 up and 4,053 down regulated genes (**Figure 3a**). KEGG analysis highlighted distinct functional patterns among the DEGs. The upregulated genes showed strong enrichment in biological pathways involving cytokine signaling and cell cycle regulation. Conversely, the downregulated genes were predominantly linked to neural signaling pathways, particularly those mediating neuroactive ligand-receptor interactions (**Figures 3b, c**).To account for tumor and immune system interaction, we employed the TISIDB dataset and identified 341 immune-related DEGs from the intersection of 998 TISIGs and 6,862 DEGs for further analysis (**Figure 3d**). Furthermore, KEGG enrichment analysis of these 341 genes revealed cytokine-cytokine receptor interaction as the most upregulated pathway. (**Figure 3e**). Taken together, identifying and analyzing of 341 immune-related DEGs linked to tumor-immune interactions provide valuable insights into TNBC immunology research and therapy.

To create a prognostic and immunotherapy response biomarker for TNBC, a risk score model was derived from 341 immune-related DEGs. Cross-validated Lasso-Cox regression was then conducted to reduce dimensionality and select the most prognostically relevant genes (**Figures 3f, g**). Furthermore, multivariate Cox regression analysis with a stepwise method was employed to establish the immune-related prognostic signature. The resulting signature consisted of four genes: CD276, Membrane Spanning 4-Domains A1 (MS4A1), Insulin Like Growth Factor Binding Protein 1 (IGFBP1) and CD200 (**Figure 3h**). And the risk score formula is as follows: 1.047*CD276-0.47*IGFBP1-0.281*MS4A1-0.548*CD200. Among them, CD276 was observed as a risk factor associated with unfavorable clinical outcomes, whereas the other three genes (MS4A1, IGFBP1, and CD200) functioned as protective factors, correlating with favorable outcomes.

### High-risk score indicates poorer prognosis in TNBC

**Figure 4:**
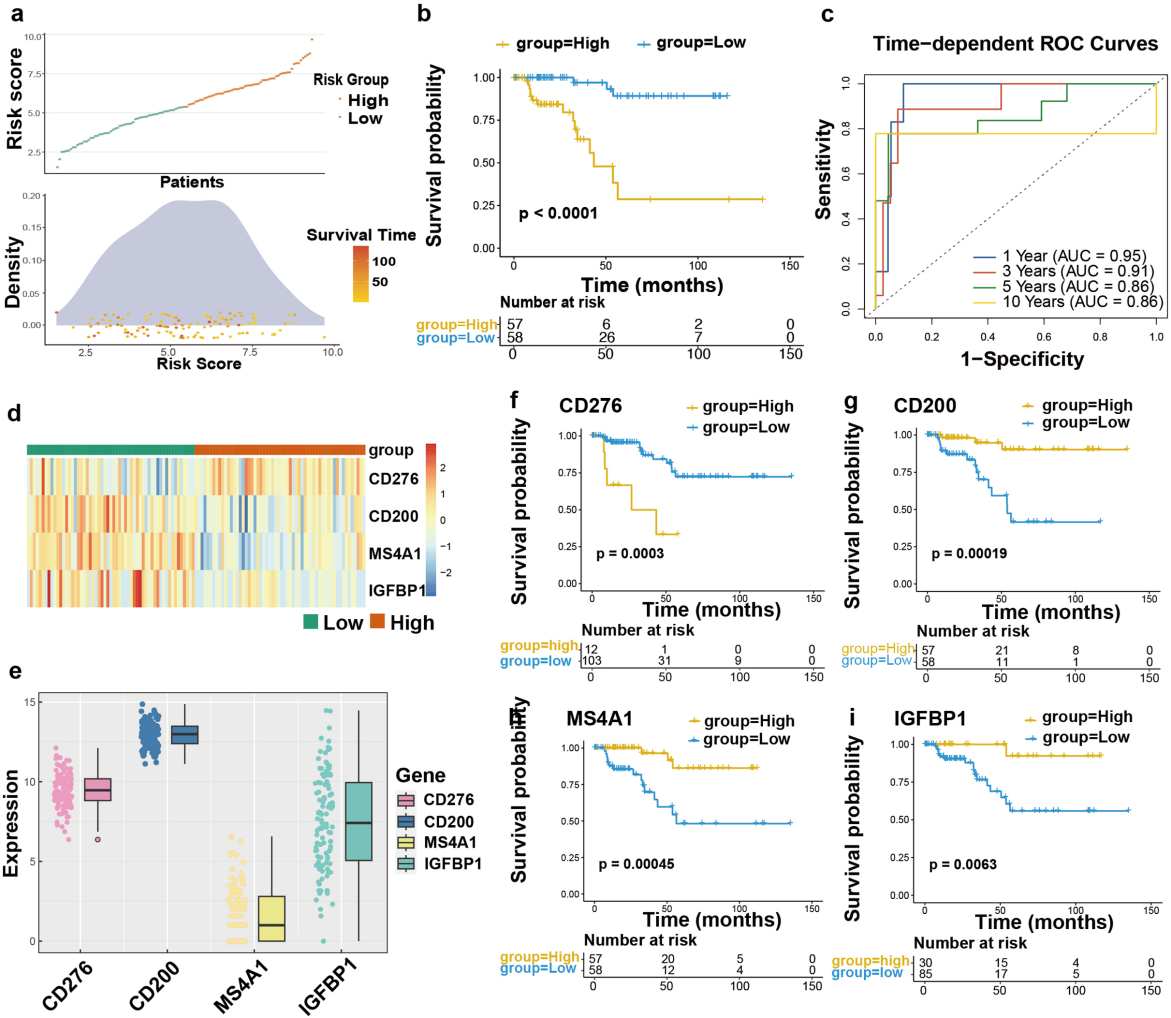
Training set in TNBC patients. **(a)** Risk score distribution and patients’ DFS density. **(b)** Survival analysis based on two groups of TCGA-TNBC patients’ DFS statues and DFS time (months, Log-rank test, HR = 0.08, 95% CI: 0.02-0.28; P<0.0001). **(c)** Time-dependent ROC curves reflect the predictive power of the prognostic signature for 1-, 3-, 5-, and 10-year outcomes. **(d, e)** Heat map and bar plot of single risk genes’ expression in the two groups. **(f-i)** Patients were grouped in accordance with every single risk genes’ expression, and survival analysis was carried out (f: HR = 0.18, 95% CI: 0.06-0.51, P = 0.0003; g: HR = 7.54, 95% CI: 2.17-26.13, P = 0.00019; h: HR = 6.81, 95% CI: 1.97-23.60, P = 0.00045; i: HR = 9.96, 95% CI: 1.32-75.38, P = 0.0063).

Based on median risk value, TCGA-TNBC cohort was categorized into two groups. Further survival analysis linked higher risk scores to reduced survival duration (**Figure 4a**). We evaluated PFS across risk groups, with high-risk cases showing reduced progression-free survival (Log-rank test, HR = 0.08, 95% CI: 0.02-0.28; P<0.0001, **Figure 4b**). Time-dependent ROC curve bolstered the reliability in forecasting outcomes for TNBC. The time-dependent ROC curve analysis demonstrated the predictive ability of the model, with the area under the curve exceeding 0.85 for the 1–10 year range. These results highlight the model’s clinical relevance and predictive accuracy across different time points (**Figure 4c**). **Figures 4d, e** illustrate the distribution of key gene expression levels. CD276 levels increased in patients deemed at high-risk, whereas protective markers (MS4A1, IGFBP1, CD200) were more abundant in low-risk cohort. We further evaluated the prognostic significance of each individual gene within the four-gene panel. Consistent with our hypotheses, elevated CD276 levels were linked to worse outcomes, whereas the other three genes correlated with more favorable outcomes (**Figures 4f-i**).

**Figure 5:**
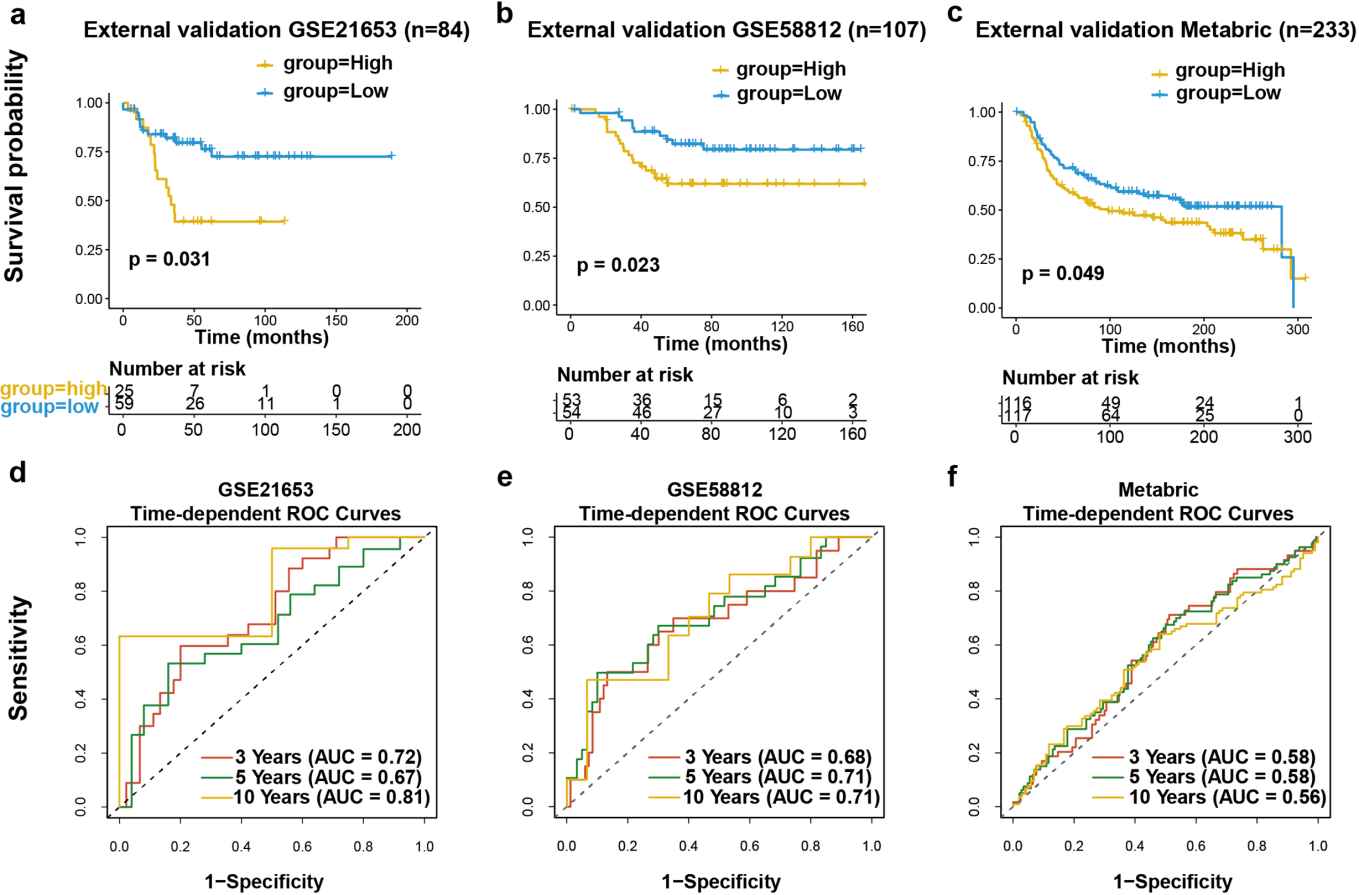
Validation in external cohort. **(a-c)** TNBC patients in GSE21653 (n = 84; HR = 0.29, 95% CI: 0.09-0.96, P = 0.031), GSE58812 (n = 107, HR = 0.42, 95% CI: 0.20-0.91, P = 0.023) and Metabric (n = 233, HR = 0.71, 95% CI: 0.50-1.00, P = 0.049) data sets were channeled into the signature, who were categorized into two groups depending on risk score. **(d-f)** Time-dependent ROC curves of above three data sets demonstrate the predictive power of 3-, 5-, and 10-year prognosis.

In order to further authenticate our discovery, we independently assessed the model using three external cohorts: GSE21653 (n=84), GSE58812 (n=107), and the Metabric cohort (n=233). Survival analysis revealed worse outcomes for high-risk cases (**Figures 5a–c**). Furthermore, the AUC values for predicting 3, 5, and 10 years survival across three independent validation cohorts consistently demonstrated the prognostic performance of our signature (**Figures 5d–f**). Moreover, to provide a clinically applicable tool for individualized prognosis prediction, we constructed a prognostic nomogram integrating the risk score and critical clinical factors via multivariable Cox proportional hazards regression with stepwise selection. Age, tumor stage, T category, N category, and the risk score were finally included into this model (**Supplementary Figure S1**). In summary, these findings confirm that the proposed signature exhibits independent predictive accuracy for estimating survival outcomes in TNBC patients.

### Prognostic signature predicts response to immunotherapy

**Figure 6:**
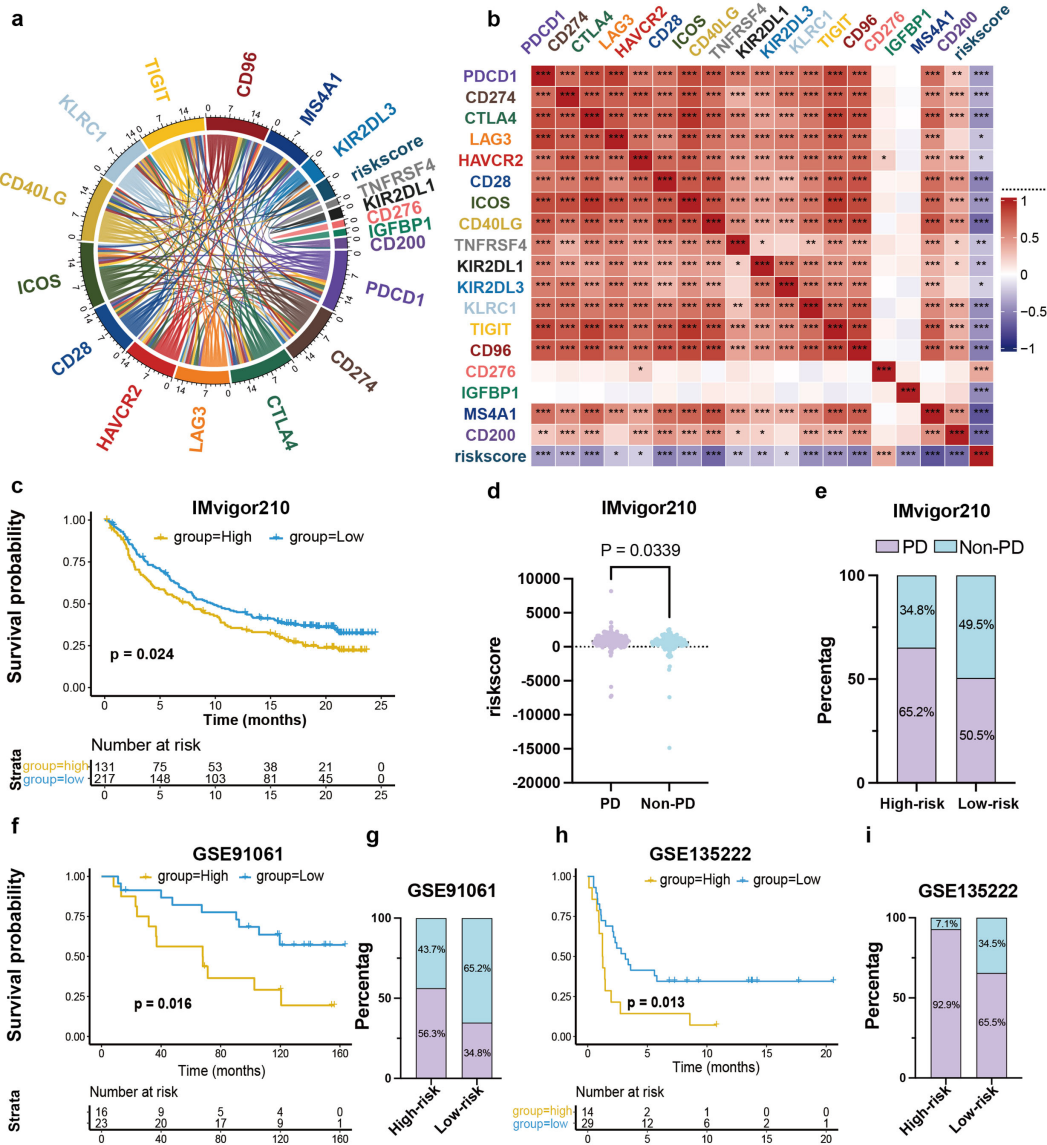
Prognostic signature predicts response to immunotherapy. **(a, b)** Correlation between major immune checkpoint-related molecules and risk score or 4 genes. **(c)** KM curve of IMvigor210 cohort (n = 348, HR = 0.74, 95% CI: 0.57-0.96, P = 0.024). **(d)** Comparison of risk scores among patients with different clinical responses to immunotherapy (IMvigor210 cohort, p = 0.0339). **(e)** Percentage of patients with PD versus non-PD after ICI therapy in risk-stratified groups in IMvigor210 cohort. **(f)** KM survival analysis of the GSE91061 (n = 43, HR = 0.36, 95% CI: 0.15-0.86, P = 0.016). **(g)** Percentage of PD versus non-PD patients after ICI therapy in risk-stratified groups of GSE91061 cohort. **(h)** Survival analysis of GSE135222 (n = 39, HR = 0.41, 95% CI: 0.20-0.84, P = 0.013). **(i)** Percentage of PD versus non-PD patients after ICI treatment in risk-stratified groups in the GSE135222 cohort.

ICIs therapy has revolutionized the clinical management of TNBC. Consequently, we examined the expression of immune checkpoint molecules and found an inverse correlation with risk score (**Figures 6a, b**, **Supplementary Figure S2**). These findings suggest our prognostic signature could predict responses to immunotherapy. Interestingly, the expression levels of MS4A1 and CD200 showed a strong relevance with the checkpoint expression, indicating their capacity in regulating the immune response, whereas the other two (CD276 and IGFBP1) exhibited modest associations.

To further evaluate the generalizability of the model for ICIs response, it was validated across multiple independent cohorts: the IMvigor210 cohort (urothelial cancer, n = 348), GSE135222 (NSCLC, n = 39) and GSE91061 (melanoma, n = 43). Analysis of KM revealed substantial reduced survival rates in high-risk patients post-immunotherapy treatment (**Figures 6c, f, h**). Elevated risk scores were correlated with reduced responses to ICIs treatment (**Figure 6d**). The at-risk population exhibited a higher proportion of progressive disease (PD) (65.2% vs. 50.5%; **Figure 6e**). The pattern persisted across the other external validation study groups: GSE91061 (56.3% vs. 34.8%; **Figure 6g**) and GSE135222 (92.9% vs. 65.5%; **Figure 6i**). The results indicate that low-risk patients stand to gain the most from immunotherapy, emphasizing this prognostic signature’s biomarker potential for identifying TNBC with highest likelihood of responding to ICIs treatment.

### Differential immune infiltration landscapes in high- vs. low-risk TNBC patients

**Figure 7:**
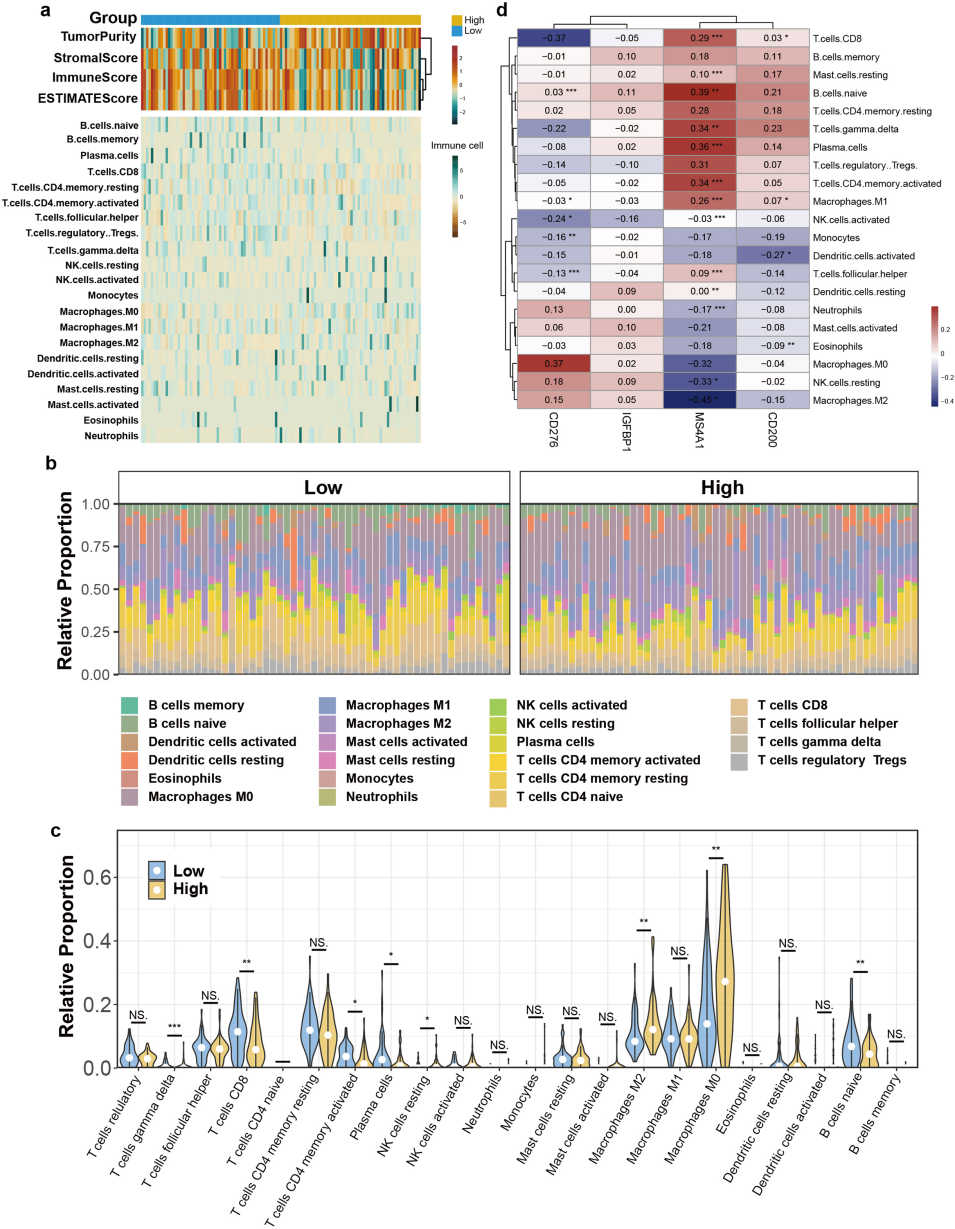
Immune infiltration Analysis. **(a)** Heatmap of results on immune cells of TME in TNBC. The data incorporates existing scores from the TIMER and MCP-counter platforms. TME-related scores are shown in the top bar. **(b)** Relative proportion of immune infiltration in risk-stratified groups. **(c)** The violin plot shows immune cell populations with significant differences by CIBERSORT. **(d)** Correlations between 4 genes and 21 immune-related cells. *P < 0.05, **P < 0.01, ***P < 0.001.

To clarify the biological principle of our prognostic signature, we characterized the TIME across the risk strata. According to the ESTIMATE algorithm, the immune score and stromal score of low-risk categories are significantly higher, and their tumor purity is lower, indicative of a highly infiltrated and potentially more immunogenic tumor. Conversely, the high-risk group exhibited elevated tumor purity and a sterile immune landscape, consistent with an “immune-excluded” phenotype (**Figure 7a**). Further deconvolution of immune cell subsets using CIBERSORT uncovered the specific cellular components driving these differences. Analysis demonstrated that higher percentages of effective CD8^+^ T, gamma delta T cells, naïve B cells, plasma cells and activated memory CD4^+^ T cells tend to clustering in low-risk group, while resting NK cells, M2 and M0 macrophages were more enriched in high-risk cohort (**Figures 7b, c**, **Supplementary Figure S3**). This clear differences in TIME provides a compelling explanation for the differential clinical outcomes and immunotherapy responses observed between the groups. Besides, we evaluated the link between risk-associated genes and immune infiltration patterns. Genes with protective roles, MS4A1, showed significant associations with the presence of anti-tumor immune cell infiltration levels. In contrast, the risk gene CD276 was positively correlated with the abundance of immunosuppressive populations (**Figure 7d**). Specifically, MS4A1 and CD200 demonstrated significant associations with levels of immune infiltration.

### Single cell analysis reveals enrichment of effector T cells and enhanced immune crosstalk in the low-risk TNBC group

**Figure 8:**
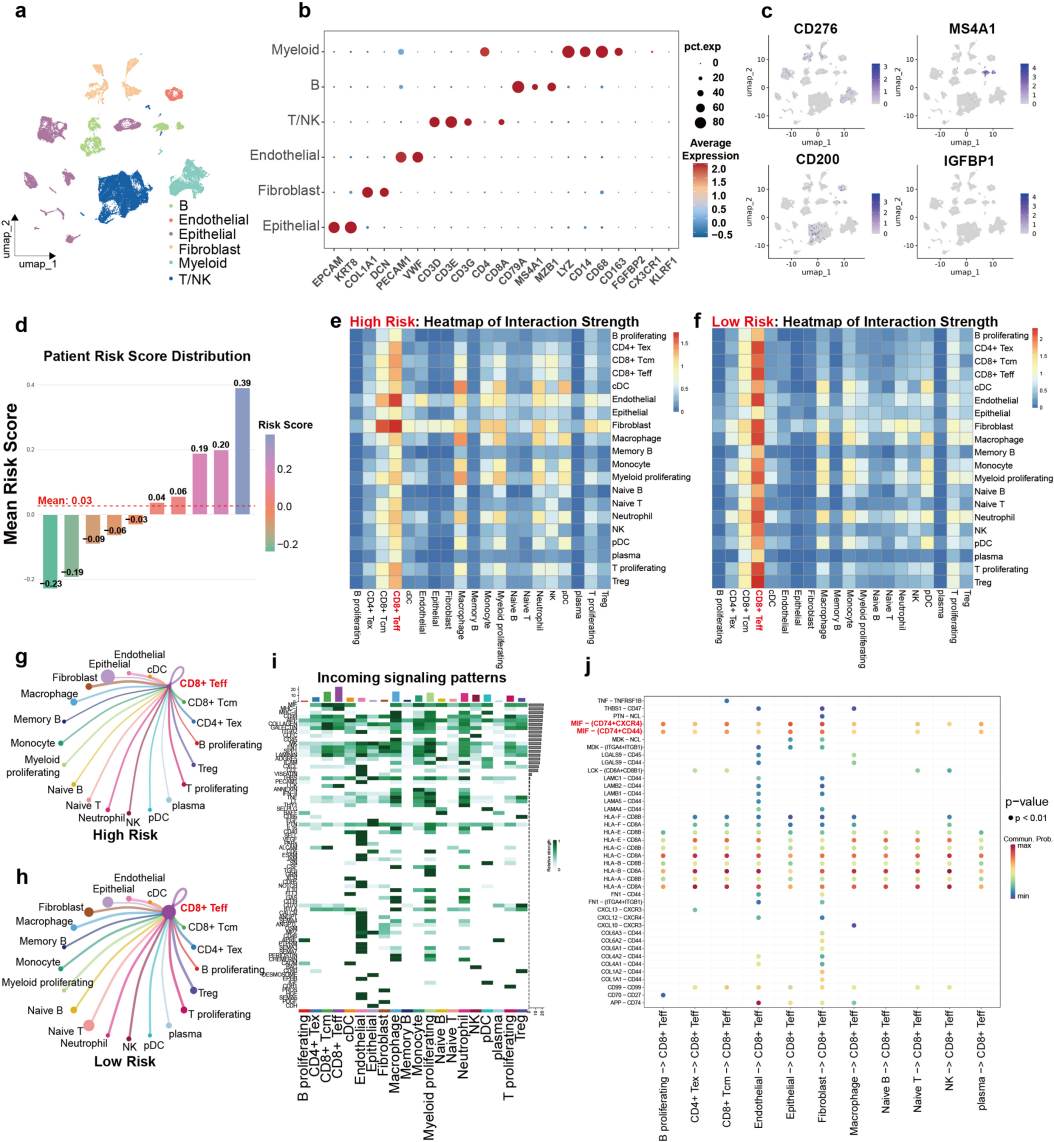
scRNA-seq reveals enrichment of effector T cells and enhanced immune crosstalk in low-risk patients. **(a)** UMAP visualization of the six identified cell types. **(b)** Marker gene for clustering. **(c)** The expression distribution of the 4 genes. **(d)** Patients’ risk score distribution calculated by z-score normalization. **(e, f)** heatmap of interaction strength between different immune cells in two risk groups. **(g, h)** Circle plot depicting the network of interactions among different cell clusters to CD8^+^ Teff cells. Edge width corresponds to communication probability; node size reflects the number of incoming signals. **(i)** Contribution of each cell type as receiver (incoming) signals in different signal patterns. **(j)** Bubble diagram of the inferred ligand-receptor interaction strengths between cell types, and enriched signaling pathways, notably the MIF- (CD74^+^CXCR4) pathways, in higher risk one.

To further clarify the functions and interactions of immune cells within the TME across risk-stratified TNBC patients, we conducted single-cell sequence analysis on TNBC cohort of GSE176078. Unsupervised clustering of the integrated dataset revealed six populations of cells, whose distribution were represented by dimensionality reduction method of UMAP (**Figure 8a**). Cell clusters were labeled based on known lineage-specific marker gene expression (**Figure 8b**). Major cell subtypes included myeloid cells, endothelial cells, fibroblasts, epithelial cells, B cells and T/NK cells. Furthermore, MS4A1 expression was largely confined to B cells, while CD200 was predominantly expressed in endothelial cells, implying their possible role in immune regulation within the TME. In contrast, CD276 and IGFBP1 were primarily expressed in epithelial cells, implying a potential engagement in modulating tumor biological behavior (**Figure 8c**, **Supplementary Figure S4**).

Each patient’s risk score was computed via z-score normalization, followed by patient classification into different risk groups (**Figure 8d**). In order to further refine the cell population and prepare for cell-to-cell communication analysis, T/NK cells, B and myeloid cells were further classified to subgroups (**Supplementary Figure S5**). We then depicted the interaction strength among various immune cell types within the TME, as visualized by heatmap generated through CellChat analysis. When it comes to low-risk group, the analysis revealed more CD8^+^ effector T cells infiltration and enhanced interaction networks between effector T cells and other immune populations (**Figures 8e-h**, **Supplementary Figure S6**). The analysis revealed pronounced ligand-receptor mediated communication between specific cell subsets. Notably, in the high-risk group, strong interactions were observed between epithelial cells/macrophage and CD8^+^ effector T cells, characterized by high level of MIF-CD74^+^CXCR4 ligand-receptor pairs (**Figures 8i, j**). In contrast, dendritic cells (DCs) and naïve B cells showed extensive incoming and outgoing signaling patterns in the low-risk group, suggesting its central role in activation of (**Supplementary Figures S7**, **S8**). These data systematically characterize the cellular crosstalk in the TNBC microenvironment across different risk strata and highlight potential immunomodulatory mechanisms that may underlie differential treatment responses.

### Risk gene expression associated with immune infiltration and tumor cell proliferation and migration

**Figure 9:**
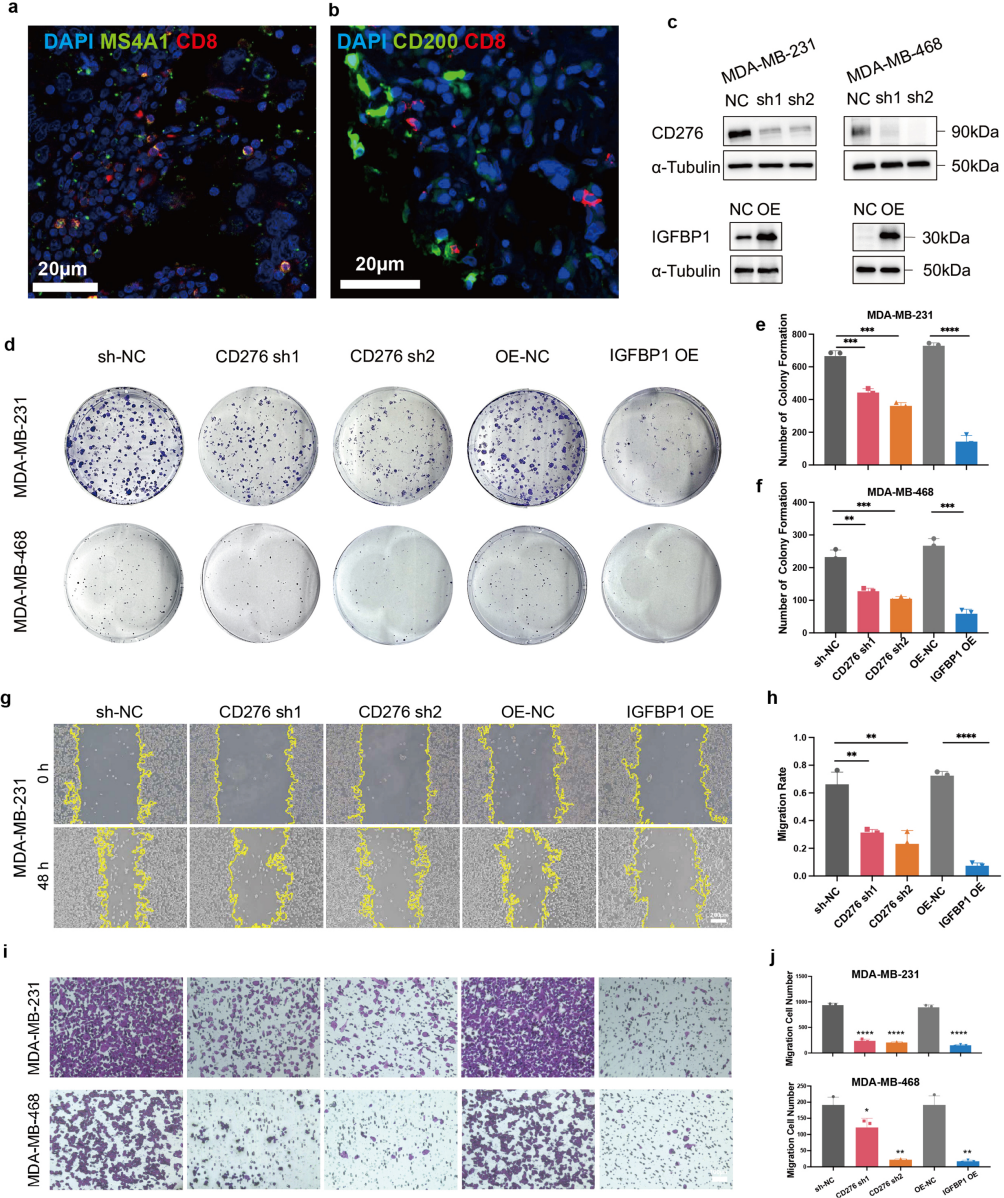
Risk gene expression mediates immune infiltration and tumor cell proliferation and migration. **(a, b)** Multiplex immunofluorescence staining of MS4A1, CD200 and CD8 in patient’s TIME. **(c)** Protein expression level of CD276 and IGFBP1 in stable cell lines and control cells. **(d-f)** Clonogenic ability and quantitative analysis of CD276-knockdown and IGFBP1-overexpressing stable cell lines. (MDA-MB-231: sh-NC vs. CD276 sh1, p = 0.0007; sh-NC vs. CD276 sh2, p = 0.0002; OE-NC vs. IGFBP1 OE, p<0.0001. MDA-MB-468: sh-NC vs. CD276 sh1, p = 0.0016; sh-NC vs. CD276 sh2, p = 0.0006; OE-NC vs. IGFBP1 OE, p = 0.0002). **(g, h)** Migratory capacity of cells and corresponding quantitative analysis. (sh-NC vs. CD276 sh1, p = 0.0028; sh-NC vs. CD276 sh2, p = 0.0048; OE-NC vs. IGFBP1 OE, p<0.0001). **(i, j)** Migratory ability of different cell lines evaluated by Transwell assay. (MDA-MB-231: sh-NC vs. CD276 sh1, sh-NC vs. CD276 sh2, OE-NC vs. IGFBP1 OE, p<0.0001. MDA-MB-468: sh-NC vs. CD276 sh1, p = 0.0315; sh-NC vs. CD276 sh2, p = 0.0058; OE-NC vs. IGFBP1 OE, p = 0.0080). *P < 0.05, **P < 0.01, ***P < 0.001, ****P < 0.0001..

Based on prior evidence indicating a strong association of MS4A1 and CD200 with immunity, we performed immunofluorescence co-staining to assess their spatial relationship with CD8+ T cells (**Figures 9a, b**). On the other hand, CD276 and IGFBP1 are highly enriched in malignant epithelium in prior analyses, we hypothesized that they may directly contribute to malignant progression. To evaluate this, we assessed their functional impact on tumor growth and migration in two kinds of human TNBC cells. In both cell lines certified by Western blot, stable CD276 knockdown and IGFBP1 overexpression were established (**Figure 9c**). CCK-8 assays illustrated reduced proliferation in CD276-KD and IGFBP1-OE cells (**Supplementary Figures S9**). The colony formation assay aligned with CCK-8 data, reinforcing the growth-inhibitory impact of CD276 silencing or IGFBP1 upregulation (**Figures 9d-f**).

To further evaluate the migratory capacities, we conducted a migrate assay and the results indicated that both of CD276-knockdown and IGFBP1-overexpression significantly impaired migration ability (**Figures 9g, h**). Consistently, the transwell assay further supported CD276 knockdown or IGFBP1 overexpression evidently inhibited cell migration (**Figures 9i, j**). These results collectively indicate that CD276 and IGFBP1 contribute to the regulation of cell migration in TNBC and may influence patient prognosis by modulating aggressive tumor behavior.

## Discussion

ICIs therapy have transformed the therapeutic paradigm for patients with TNBC (14). Despite the revolutionary progress, many patients still show inherent or acquired resistance to ICIs treatment (36, 37). Although some biomarkers, like PD-L1 expression, have been established for patient selection, their predictive utility remains imperfect due to methodological inconsistencies and limited sensitivity in specific clinical contexts (16, 38). Moreover, emerging evidence suggests that TMB, while approved as a pan-cancer biomarker, only correlates with a modest response rate in breast cancer, emphasizing the imperative requirement for broader biomarkers to effectively categorize patients eligible for ICIs (39–41). In this study, based on our findings regarding the correlation between immune infiltration and survival in an internal cohort, we developed a four-gene prognostic signature for ICIs treatment efficacy in TNBC by integrating evaluation of both tumor-related characteristics and immune system features.

Here, we integrated and refined TCGA sourced tumor-related DEGs with genes from TISIDB related to immune to ultimately establish a four-gene prognostic signature comprising CD276, MS4A1, IGFBP1 and CD200, which has the ability to reflect ICIs responses and survival outcomes. This signature demonstrated its’ capacity in predicting both short- and long-term survival, suggesting its potential clinical application for risk stratification in TNBC. Notably, risk score, independent of other clinical characteristics, has been proved as a prognostic factor, reinforcing its value as a complementary tool in current prognostic systems.

What’s more, our results also indicate a link between low-risk groups and a higher immunogenic TME. The component and state of immune cells within TME are well-established determinants of response to ICI therapy (42–45). The immunity-tumor communication is pivotal in tumor progression and therapeutic outcomes (46). Extensive research has consistently demonstrated that a high infiltration of cytotoxic CD8^+^ T cells and CD4^+^ T helper1 cells is a prerequisite for clinical benefit from ICIs (47–49). These cells are the main anti-tumor force, and their presence indicates a baseline capacity for tumor recognition that can be reinvigorated by checkpoint blockade. A key finding of our study, elucidated in **Figure 7**, is the ability of our four-gene signature to accurately capture the structure of the TIME. The enriched presence of CD8^+^ effector T cells and enhanced interaction networks in the low-risk cohort, align with this paradigm of immune activation. The strong correlation between the protective genes (MS4A1/CD200) and beneficial immune infiltrates underlays their potential involvement in recruiting or maintaining these populations. For instance, MS4A1, as a B-cell marker, directly reflects the presence of a B-cell lineage, emerging evidence uncovers the underestimated function of B cells within TLSs (50). The density of plasma and B memory cells has been linked to superior outcomes following ICI treatment, potentially through antigen presentation, antibody production and immune-modulatory cytokine secretion, highlighting the importance of a coordinated humoral immune response (51). Our results also showed that dendritic cells (DCs) and naïve B cells exhibited extensive incoming and outgoing signaling patterns in the low-risk group. Overall, our signature clearly categorized patients into various risk strata and demonstrated the capability to predict the level of immune activation within patients—particularly the infiltration of effector T cells and its function—thereby offering an indicators for survival and response to ICI therapy predicting.

At the biological level, each gene in the signature contributes distinctively in tumor biology and immune regulation. CD276 (also known as B7-H3), identified in our model as a risk factor, belongs to the B7 family of immune molecules (52). Consistent with our finding, CD276 is frequently overexpressed on various cancer cells and even on tumor-associated vasculature, while its receptor remain somewhat elusive (53). Numerous studies have consistently reported its contribution in promoting immune evasion. It inhibits T cell activity and reduces cytokine production, thereby fostering an immunosuppressive TME (54). Beyond its immunomodulatory functions, CD276 also exerts non-immune oncogenic effects by enhancing tumor cell migration, invasion (55). This oncogenic role was further confirmed in our *in vitro* functional experiments. This dual role in promoting both immune evasion and aggressive tumor behavior aligns with our results, where high CD276 levels associated with a suppressive immune landscape and adverse prognosis. MS4A1 (CD20), a well-established surface marker for B cells, functioned as a protective factor in our signature. Its role is intrinsically linked to the presence and function of B lymphocytes within the TME (56). Contrary to the historical focus solely on T cells, recent evidence highlights that B cells are strong positive predictors for response of immunotherapy and OS among multiple tumors, including TNBC (57). CD20^+^ B cells may improve anti-tumor ability by presenting antigens to T cells as well as releasing inflammatory cytokines (58). Our observation that the low-risk group exhibits greater T-B cell crosstalk, which correlates with a favorable immune contexture, underscores its critical function in anti-tumor ability. IGFBP1 is a receptor for the biological utilization of IGF. Its role in cancer is complex and vague (59). For instance, in hepatocellular carcinoma, IGFBP1 acts as an inhibitor of IGF-1R pathway signaling. High IGFBP1 expression correlated with better OS, and *in vitro* research showed that it suppressed cell growth and induced apoptosis via caspase-3 activation (60). In our study, it was also identified as a protective factor and we observed that IGFBP1 significantly inhibited TNBC cell growth and migration. The protective role of CD200 observed in our study reveals its context-dependent and complex functions within the TIME, challenging the conventional view of CD200 as primarily immunosuppressive. While the CD200-CD200R axis is known to deliver inhibitory signals that weaken antigen-presenting cell and T cell activity, our findings align with emerging evidence that CD200 can also exert tumor-restraining effects (61). Two mechanisms may explain this protection: first, the CD200-CD200R pathway may help maintain immune homeostasis by curbing excessive inflammation, thereby preventing inadvertent tumor promotion; second, its cellular source is critical—our single-cell analysis indicates predominant endothelial expression of CD200, which may regulate immune cell trafficking and adhesion, thereby shaping the quality of anti-tumor immunity (62–64). Additionally, recent studies highlight a subset of CD200^+^ cytotoxic T lymphocytes with enhanced activity that contribute to anti-PD-1/PD-L1 efficacy (63). Thus, the net role of CD200 in TNBC likely reflects a balance between direct immunosuppressive signaling and its broader function in fostering an organized, homeostatic, and functional TIME. Our data emphasize the latter as dominant, underscoring the importance of cellular origin and microenvironmental context in interpreting immune checkpoint biology.

Interestingly, high-risk patients exhibited pronounced MIF-mediated reciprocal actions among macrophages/epithelial cells and CD8 positive T, suggesting a potential mechanism of T cell dysfunction in immune-cold tumors. This specific ligand–receptor cross-talk is primarily through the MIF–CD74/CXCR4 axis. The sustained MIF signaling may promote CD8^+^ T cell exhaustion, also improve impair effector functions, ultimately assisting immune escape (65, 66). The results align with existing research implicating MIF as an immunosuppressive microenvironment factor and emphasize its capacity to reverse T cell inhibition in poorly infiltrated tumors (67).

Except for those insights, this study still has imperfections. First, prospective cohort studies are needed to provide further validation of the model. Second, the precisely potential mechanisms about how MS4A1 and CD200 play a role in immune activity and patient prognosis need further investigation, *in vivo* B-cell depletion and reconstitution experiments and CD200-blocking antibodies or endothelial-specific knockout models are need to perform in the future. Finally, although *in vitro* researches have showed that CD276 and IGFBP1 clearly influence the proliferation and migration, further experimental evidence and the pathology validation in a large, independent cohort of TNBC tissues is waiting for collecting, so that the potential molecular mechanisms can be revealed.

In conclusion, we have established a novel immune-related signature that effectively predicts prognosis and immune landscape in TNBC patients. Our work highlights the interplay between tumor-intrinsic properties and immune modulation, providing a mechanistic basis for the differential clinical outcomes observed between risk groups. These findings not only offer a new tool for patient stratification but also characterize the immune landscape across different risk-stratified groups.

## Data Availability

The original contributions presented in the study are included in the article/**Supplementary Material**. Further inquiries can be directed to the corresponding authors.
