# Supplementary material for: Multi-omics profiling unravel the immune landscape diversity by prognostic signatures of immunotherapy response in triple-negative breast cancer
Source: Front Immunol. 2025 Dec 18;16:1735893. doi: 10.3389/fimmu.2025.1735893 (PMC12756148; doi:10.3389/fimmu.2025.1735893)
Supplement: Supplementary file 1 [file DataSheet1.docx]

**Supplementary Materials**

**Multi-Omic Profiling Unravel the Immune Landscape Diversity by Prognostic Signatures of Immunotherapy Response in Triple-negative Breast Cancer**

*Rong Chai^1^, Ziting Zhang^1^, Zheng Gong^1^, Qi Li^1*^, Chunyan Dong^1*^*

^1^Department of Oncology, Shanghai East Hospital, School of Medicine, Tongji University, Shanghai 200092, China.

***Corresponding author:**

Chunyan Dong

Department of Oncology, Shanghai East Hospital, School of Medicine, Tongji University, Shanghai 200092, China.

Email: [cy_dong@tongji.edu.cn](mailto:cy_dong@tongji.edu.cn)

Telephone number: 13370029736

ORCIDs: https://orcid.org/0000-0003-2392-3185

Qi Li

Department of Oncology, Shanghai East Hospital, School of Medicine, Tongji University, Shanghai 200092, China.

Email: [qf.1007@163.com](mailto:qf.1007@163.com)


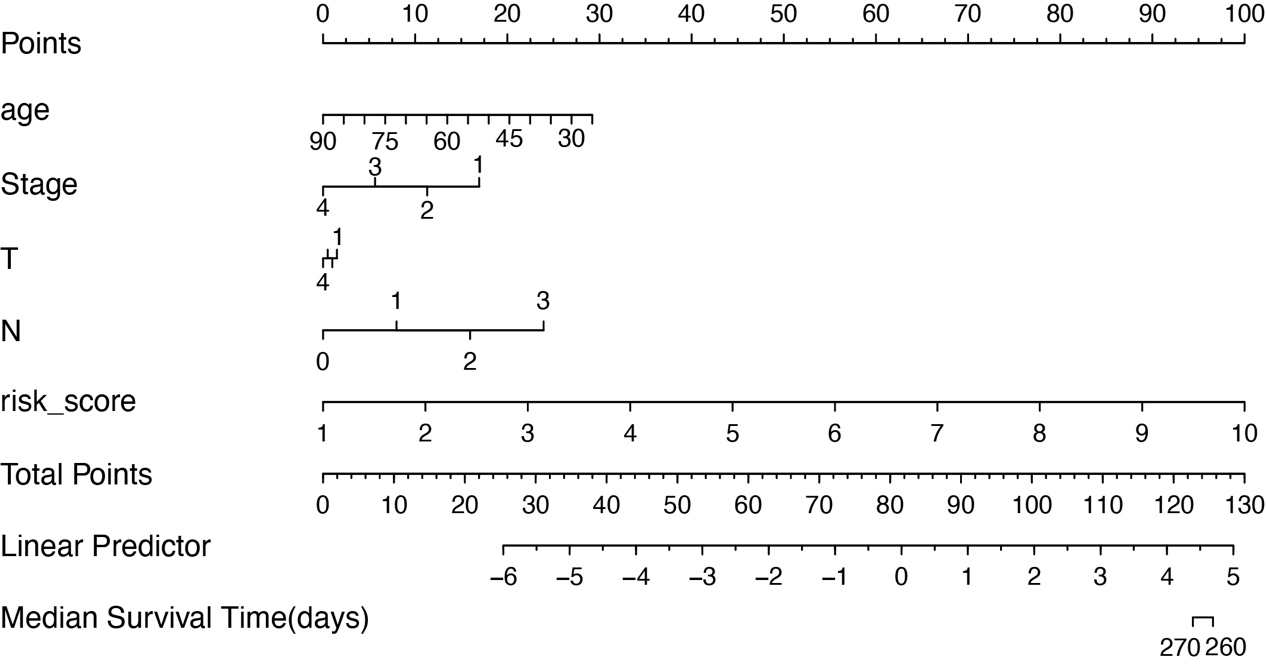


**Fig. S1** Construction of the nomogram integrating the age, stage, T,N-stage clinical stage and risk score.


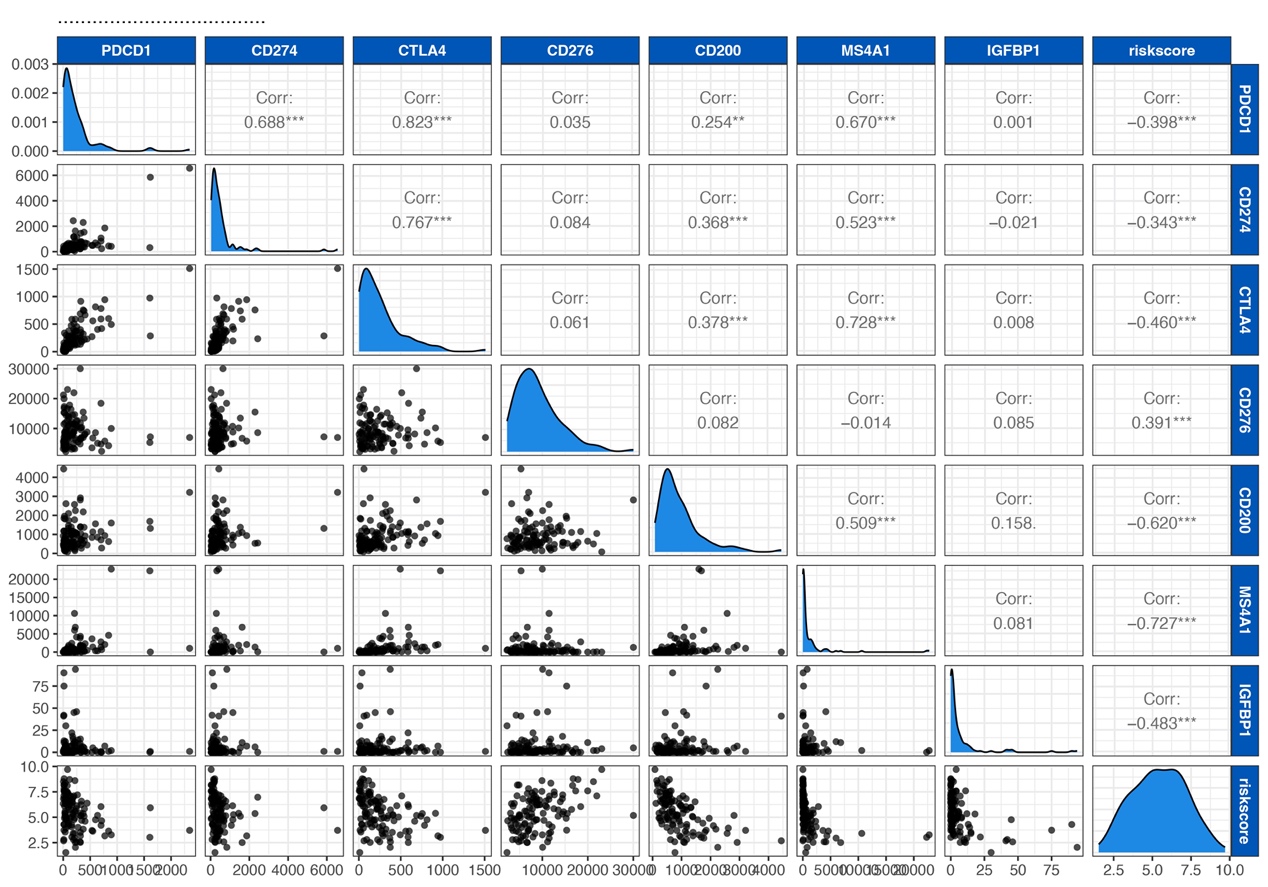


**Fig. S2** Correlation between major immune checkpoint-related molecules and risk score or 4 genes.


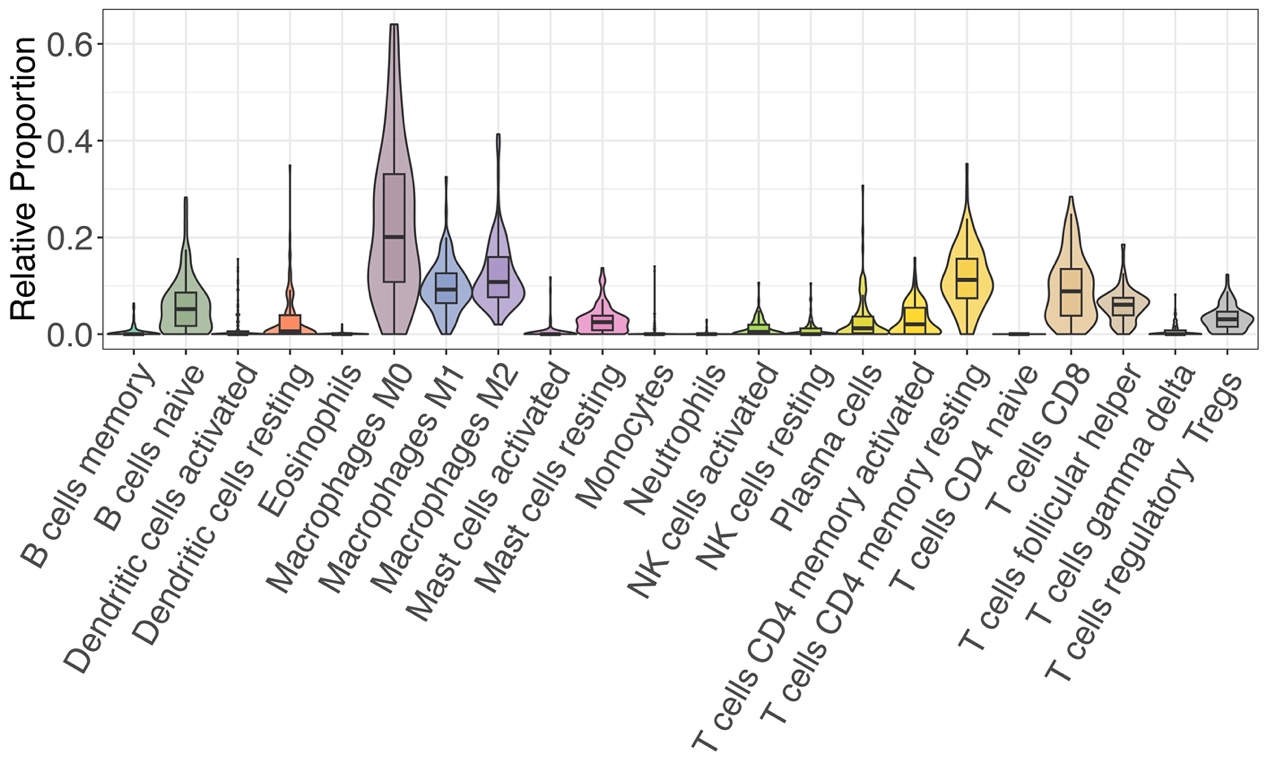


**Fig. S3** Relative proportion of immune infiltration in all patients by CIBERSORT algorithm.


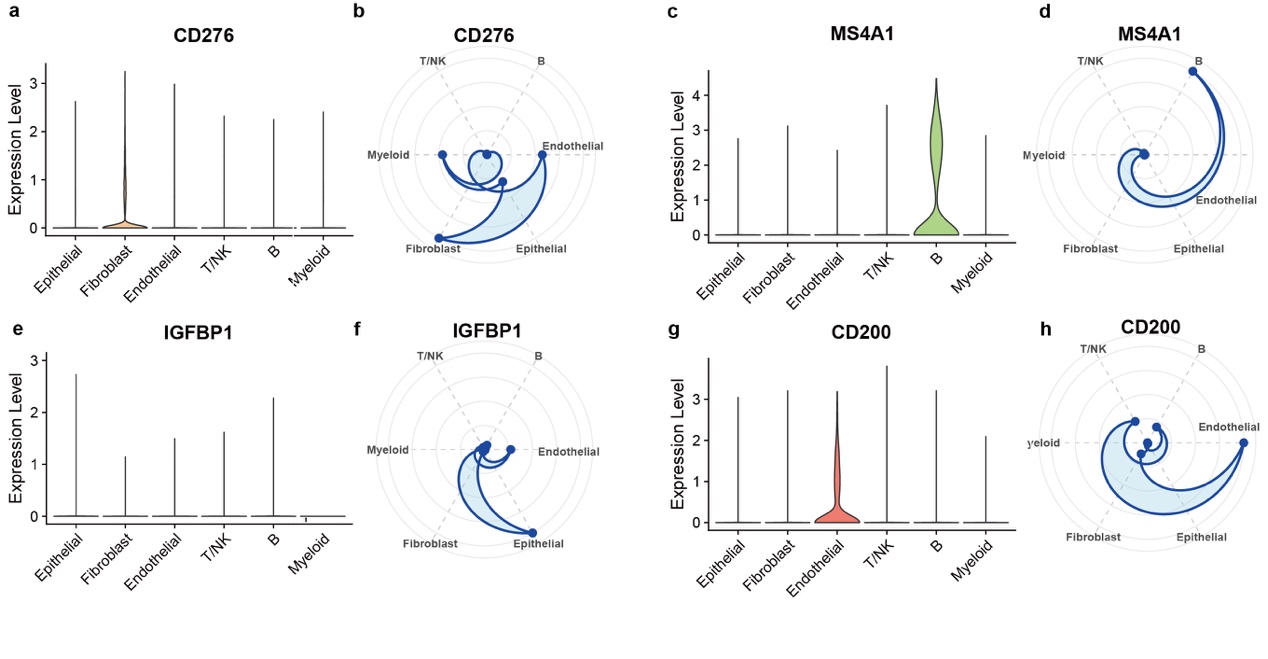


**Fig. S4** Expression of 4 genes in different cells cluster. **a, b** Expression level of CD276 among different cells by Violin Plot and Radar Chart. **c, d** Expression level of MS4A1 among different cells. **e, f** Expression level of IGFBP1 among different cells. **g, h** Expression level of CD200 among different cells.


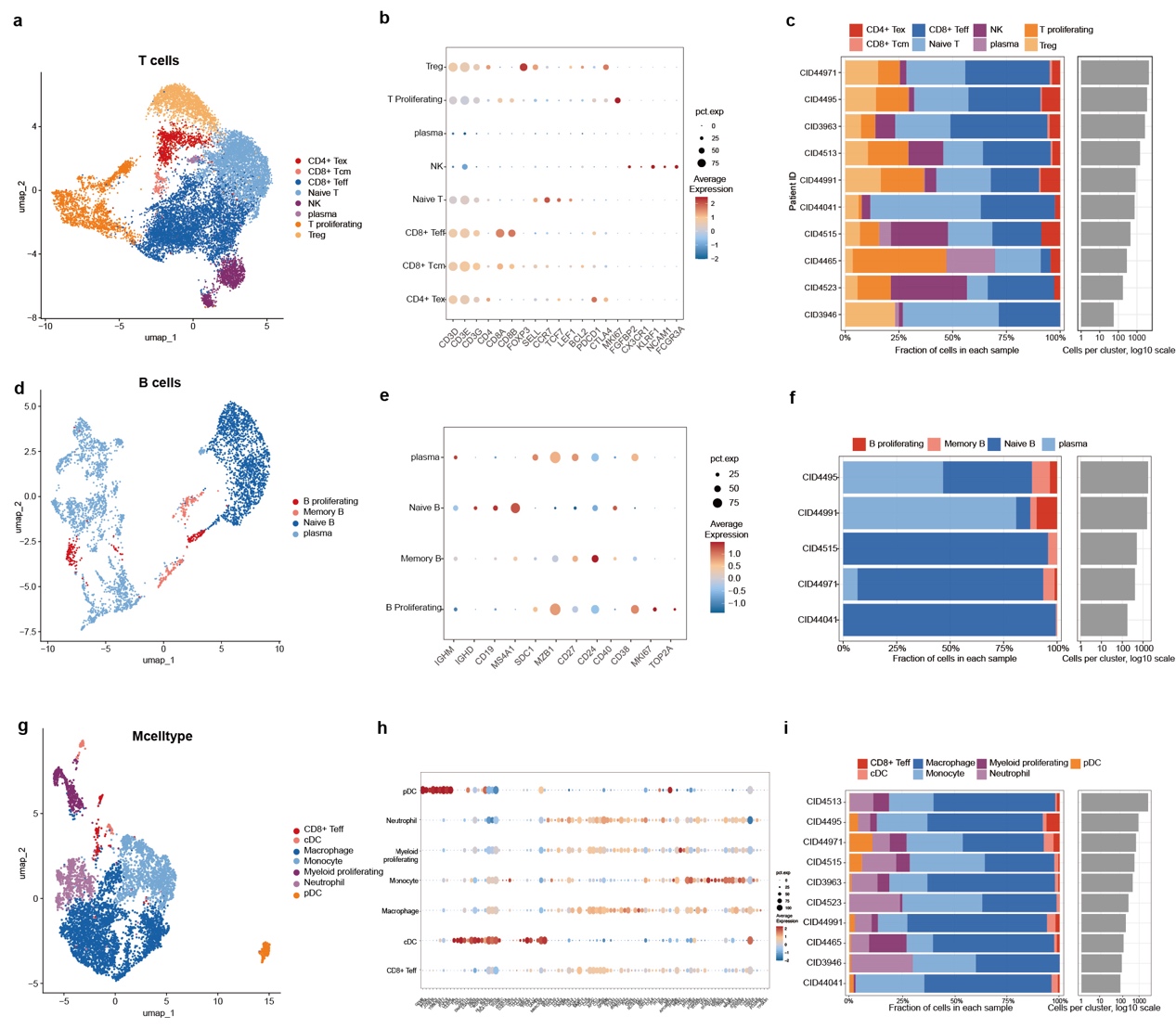


**Fig. S5** Classification of immune cell subsets. **a, d, g** UMAP visualization of the T cells , B cells, and the myeloid subsets. **b, e, h** Bubble plot showing the marker genes used for UMAP cluster annotation. Proportion of different T cell subsets in each sample. **c, f, i** Proportion of different T cell, B cell and myeloid subsets in each sample.


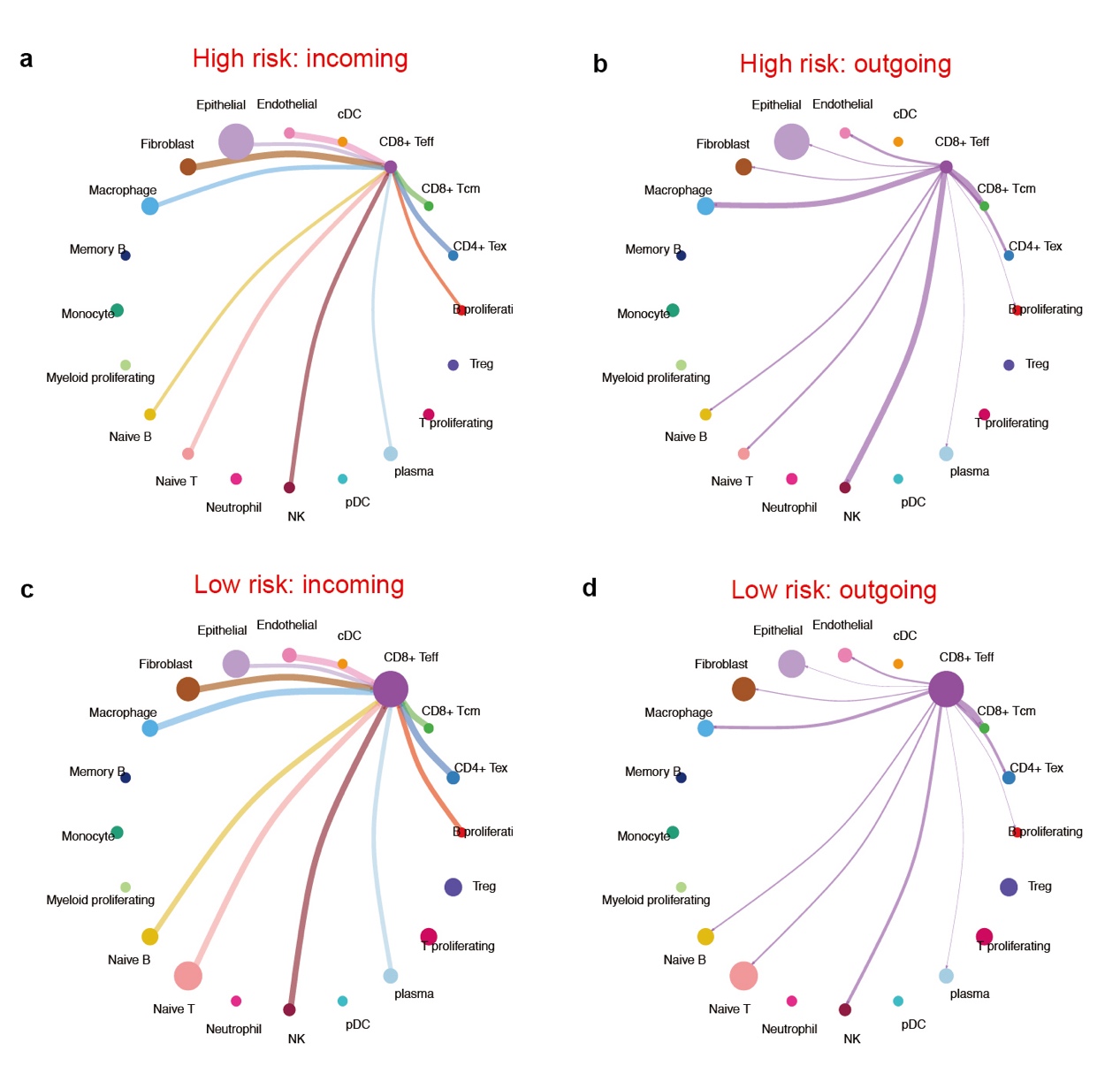


**Fig. S6** Cellchat between specific cell clusters. **a, b** The incoming and outgoing cellchat between CD8^+^ Teff cells and other cells in High-risk group. **c, d** The incoming and outgoing cellchat between CD8^+^ Teff cells and other cells in Low-risk group.


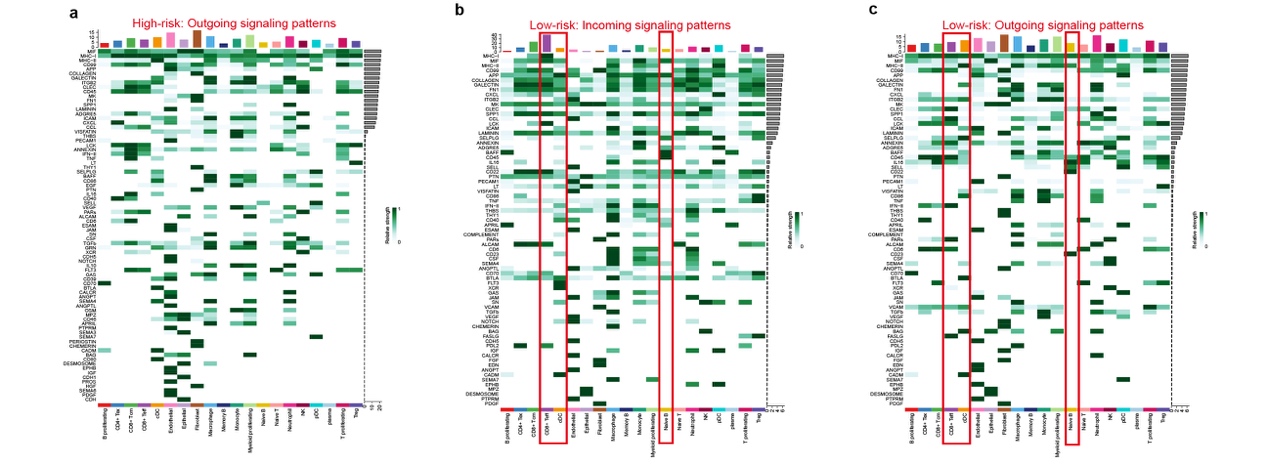


**Fig. S7** Heatmap of signaling patterns. **a** Outgoing signaling patterns of different immune cells in High-risk group. **b** Incoming signaling patterns of different immune cells in Low-risk group. **c** Outgoing signaling patterns of different immune cells in Low-risk group.


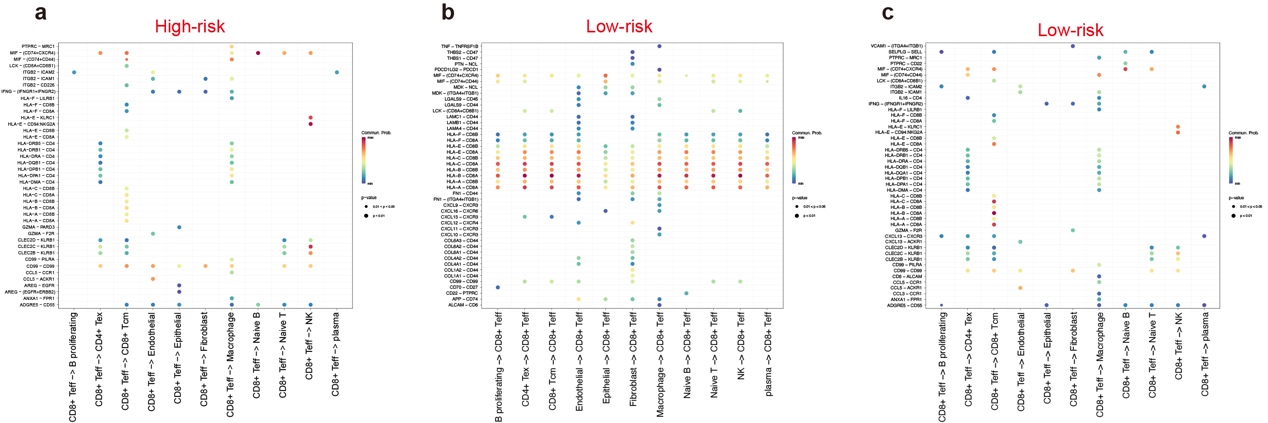


**Fig. S8** Differentially enriched signaling pathways in high- versus low-risk groups.


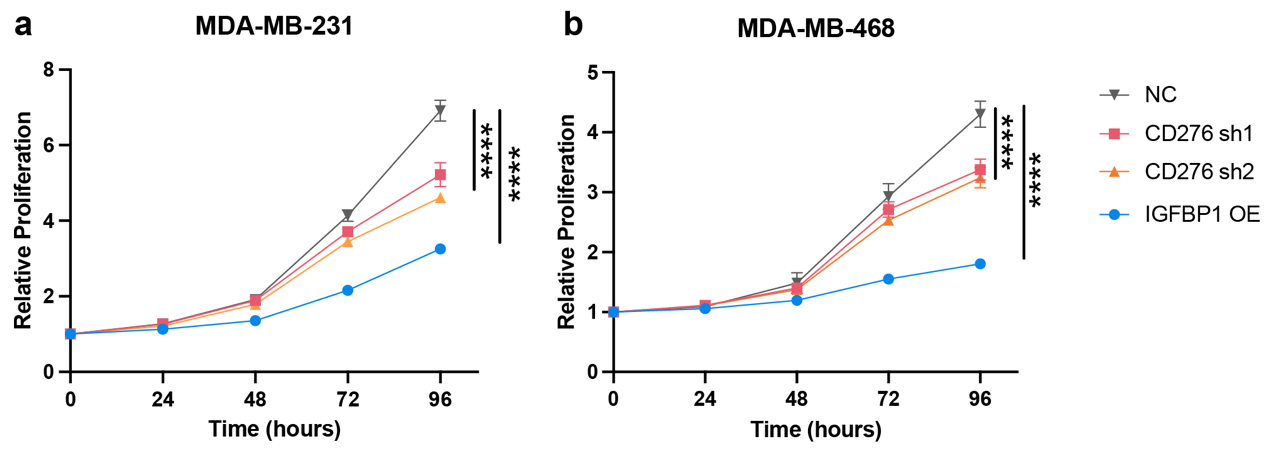


**Fig. S9** Cell proliferation was evaluated using the CCK8 assay in MDA-MB-231 (**a**) and MDA-MB-468 (**b**).
